# Supplementary material for: Confidence and insight into working memories are shaped by attention and recent performance
Source: J Cogn Neurosci. Author manuscript; Available in PMC 2025 Jul 21. (PMC7617916; doi:10.1162/jocn_a_02326)
Supplement: Supplementary Materials [file EMS207239-supplement-Supplementary_Materials.pdf]

Appendices

Simulation Methods:

For all simulations, a standard procedure was used to generate artificial participant data. A mixture model was fitted to the participant data from experiment 1 (Bays, Catalao & Husain, 2009). This model decomposes participant response distributions as a mixture of component distributions that reflect different processes. The model fit gives rise to three parameters: *Kappa*, the concentration of the best-fitting von mises distribution that describes response variability; *Pn*, the probability of non-target responses, and; Pu, the probability of random responses (a uniform distribution that captures random guessing). This model was fit separately for cued and neutral trials per participant.

The across-subject mean and standard error *Kappa* parameter for each condition were used as parameters for a normal distribution, from which we randomly sampled to obtain a simulated precision of the response error distribution for neutral and cued trials. Using these simulated concentration parameters, we generated a response error distribution for neutral and cued trials with mean 0 and a concentration parameter dependent on the sampled value.

These simulations were performed under the null hypothesis that a perfect observer has no insight into the single-trial noise of their mnemonic representations, and thus no insight into their single-trial error - an optimal agent with perfect knowledge of the inaccuracy of their memory would adjust their response to account for this, and thus make a response with zero error on every trial. Instead, a perfect observer may have perfect insight into their across-trial error distribution, and thus their confidence estimate would be a noisy (random) sampling of this error distribution. To simulate confidence under this null distribution, we randomly sampled confidence on each trial from the same underlying distribution as response error, taking the absolute value of this sampled error (as you cannot have negative confidence).

For each simulation, an experimental dataset was simulated. We generated twenty artificial participants per simulated dataset, with 128 trials per cue condition. Ten-thousand simulated datasets were created using this procedure, allowing us to build up a simulated null distribution of the relevant test statistic under the null hypothesis that confidence estimates are generated with no insight into single-trial memory error.

Permutation methods

An alternative method to investigate this null belief is to use permutations. Rather than simulating datasets, the experimental data are randomly shuffled (permuted) to preserve individual subject response error and confidence distributions, but remove any relationship between single-trial performance and confidence. This, across permutations, builds up a distribution of the relevant test statistic for the experimental dataset, reflecting the distribution of test statistics that arise in the data due to random chance. The proportion of test statistics, in this permuted null distribution, larger than the observed experimental effect, reflects the p-value for our observed results.

Appendix 1 – Observed correlations between error and confidence do not arise due to chance

We show evidence that subjective confidence judgements are associated with response error, suggesting insight into performance on the individual trial. However, across trial relationships could arise not due to insight into the single-trial response error, but instead from perfect insight into the across trial response error distribution. In such a model, individuals would not have insight into their individual trial WM error as an observer with insight into the exact error in their WM representation would alter their response to account for this, leading to zero error. Instead, an individual may have perfect knowledge of the across-trial error distribution, but no insight on the individual trial. In such a scenario, confidence estimates would be generated by randomly sampling from the across-trial error distribution, to generate a subjective uncertainty in each trial. The across trial confidence distribution would then match identically the response error distribution.

To test for such an explanation for our observed results, we ran a permutation-based analysis on the experimental data gathered in experiment 1. For each subject, confidence was shuffled across trials for each cue condition separately. Spearman's rank correlation was calculated for the relationship between response error and this shuffled confidence, separately for each cue condition. This was repeated 10,000 times for each subject, generating a null distribution of the spearman's rank correlation between response error and confidence for each participant and condition in the task (see Appendix Figure 1A for an example null distribution for a participant).

To test whether the observed correlation was reliably larger than the relationship expected under the null (the mean correlation of the permuted null distribution), a paired-samples t-test was performed, across subjects, on the fisher-transformed correlation coefficients, separately for each cue condition. This test was significant for both neutral trials ($t(19) = 7.27$, $p = 3.39 \times 10^{-7}$) and cued trials ($t(19)$ 6.29, $p = 2.43 \times 10^{-6}$). This suggests that participants reported confidence across trials was related to response error, and integrated information about performance at the level of the individual trial. Individual participant correlation coefficients are visualised in Appendix figure 1B, along with summary statistics of the generated null distributions.

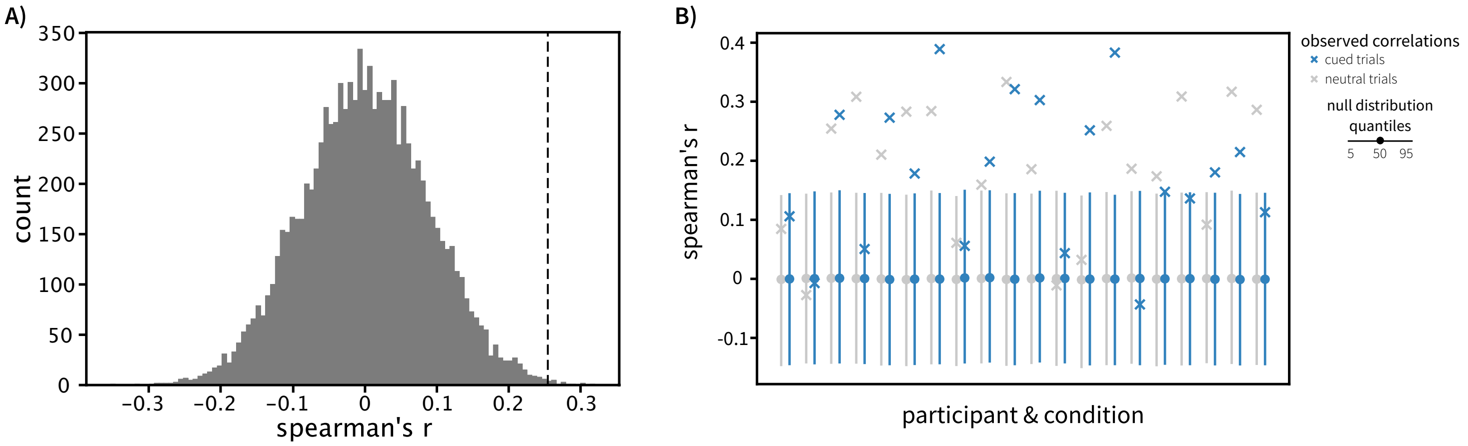

Appendix Figure 1 - A) Histogram visualising an example permuted null distribution of correlation coefficients. Dashed black line shows the observed correlation for this participant. B) Visualisation of the observed correlations for each subject, and the relevant permuted null distribution. Solid circles show the expected (mean) permuted spearman's rank correlation for each participant and condition. Solid lines represent the 5th to 95th percentile of the permuted null distributions. Crosses reflect the observed spearman's rank correlation, separately for each condition and participant. Blue marks reflect the relevant statistics for cued trials, with grey representing neutral trials.

Appendix 2 – Observed random effects do not arise due to perfect insight to across-trial error distributions

I. Permutation

To determine whether the result of our random-effects analysis (showing that the relationship between response error and confidence is modulated by cue type) was due to chance, we conducted a permutation-based control analysis. In each permutation, we shuffled the reported confidence of each participant, separately per participant and condition, to preserve the individual subjects' confidence distributions but unlink error and confidence at the level of the individual trial. We then ran the random effects analysis, implementing the same single-subject GLM as in the main analysis, and conducting a two-sided t-test on the interaction term across participants. This t-statistic for the interaction was generated for each of the 10,000 permutations that were implemented on the data. The significance of the observed, experimental results was calculated by the proportion of permutations giving rise to a t-value larger than we observed in analysis of the experimental data (see Appendix Figure 2A). The p-value for our observed t-value was 0.0034 (only 34 of 10,000 permutations gave rise to a larger t-value than our experimental result).

II. Simulation

A simulation was run to explore the range of effects that could arise under the previously stated null belief, to determine whether simulated results are concordant with the findings of our permutation-based control analysis. We simulated 10,000 experimental datasets, implementing our random-effects analysis on each experimental dataset. A distribution of the resulting test statistics (see Appendix Figure 2B) was generated, and our experimental analysis result was compared to this distribution. We found that only 32 of 10,000 simulated datasets gave rise to a t-value larger than our experimental analysis (p = 0.0032).

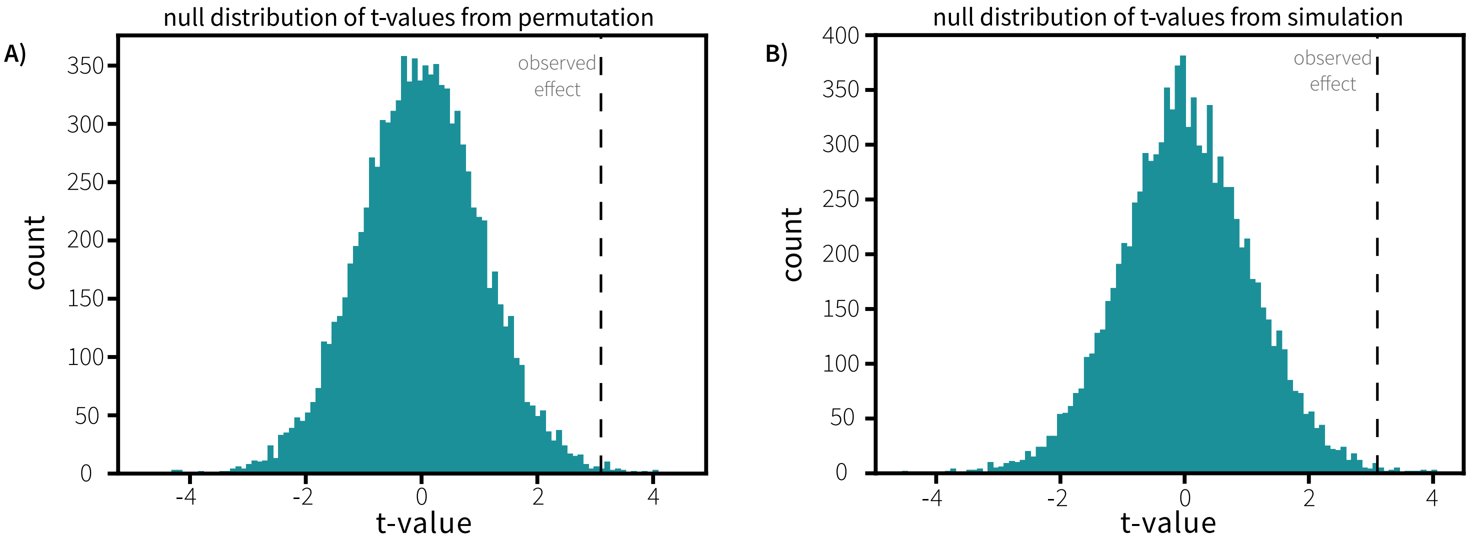

Appendix Figure 2 - A) Distribution of t-values from permutation-based control analysis of the random effects. B) Distribution of t-values from control analysis using simulation of random effects across simulated datasets. Dashed lines in both panels represent the empirically-observed t-value for the random-effects analysis.

Appendix 3 – Observed linear mixed-effects do not arise due to perfect insight into across-trial error distributions.

I. Permutation

To determine whether the result of our linear mixed-effects analysis (showing that the relationship between response error and confidence is modulated by cue type) was due to chance, we conducted a permutation-based control analysis. In each permutation, we shuffled the reported confidence of each participant, separately per participant and condition, to preserve the individual subjects' confidence distributions but unlink error and confidence at the level of the individual trial. We then ran the linear mixed-effects analysis, implementing the same model structure as in the main analysis. The t-value for the interaction term of this analysis was then generated for 10,000 permutations of the data. The significance of the observed, experimental results can be assessed by the proportion of permutations giving rise to a t-value larger than we observed in analysis of the experimental data (see Appendix Figure 3A). The p-value for our observed t-value was 0.0014 (only 14 of 10,000 permutations gave rise to a larger t-value than our experimental result).

II. Simulation

A simulation was run to explore the range of effects that could arise under the previously stated null belief, to determine whether simulated results are concordant with the findings of our permutation-based control analysis. We simulated 10,000 experimental datasets, implementing our linear mixed-effects model on each experimental dataset. A distribution of the resulting test statistics (see Appendix Figure 3B) was generated, and our experimental analysis result was compared to this distribution. We found that only 12 of 10,000 simulated datasets gave rise to a t-value larger than our experimental analysis (p = 0.0012).

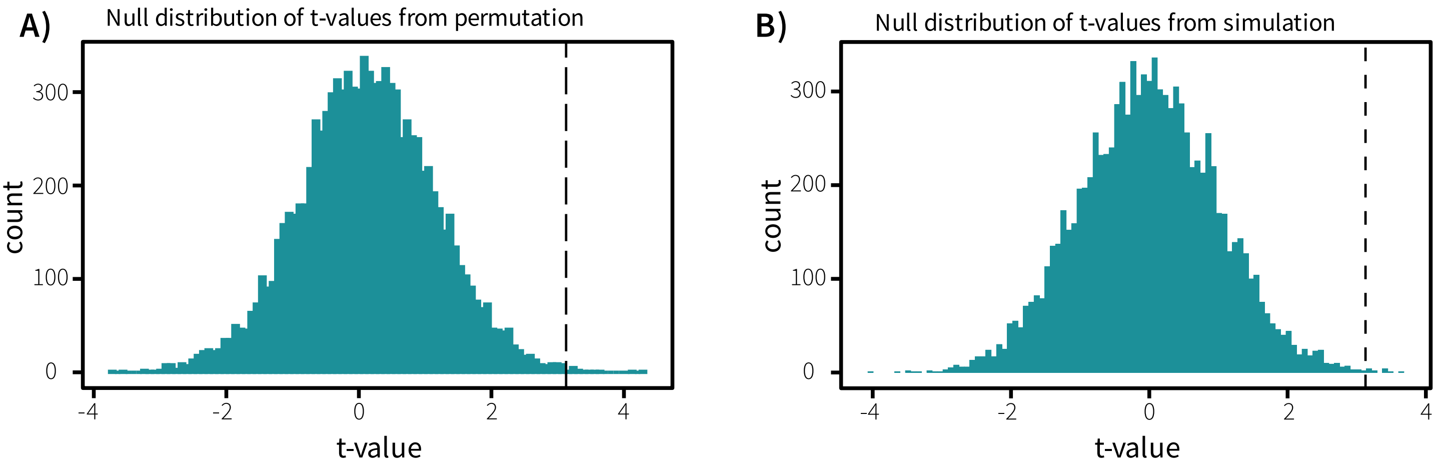

Appendix Figure 3 - A) Distribution of t-values from permutation-based control analysis of the linear mixed-effects analysis. B) Distribution of t-values from control analysis using simulation of linear mixed-effects across simulated datasets. Dashed lines in both panels represent the empirically-observed t-value for the random-effects analysis.

Appendix 4 – cue-induced alpha lateralisation median split by behaviour

Model-based analysis of the cue response period highlighted distinct time windows of alpha lateralisation that are predictive of error and confidence in working-memory responses. For illustrative purposes, we provide an alternative visualisation of this finding. For neutral (left column) and cued (right column) trials separately, we calculated a within-participant median split on response error (top row) and confidence (bottom row), then averaged across participants. We plot these median splits in each panel, with black lines reflecting the across-participant average of the within-participant difference.

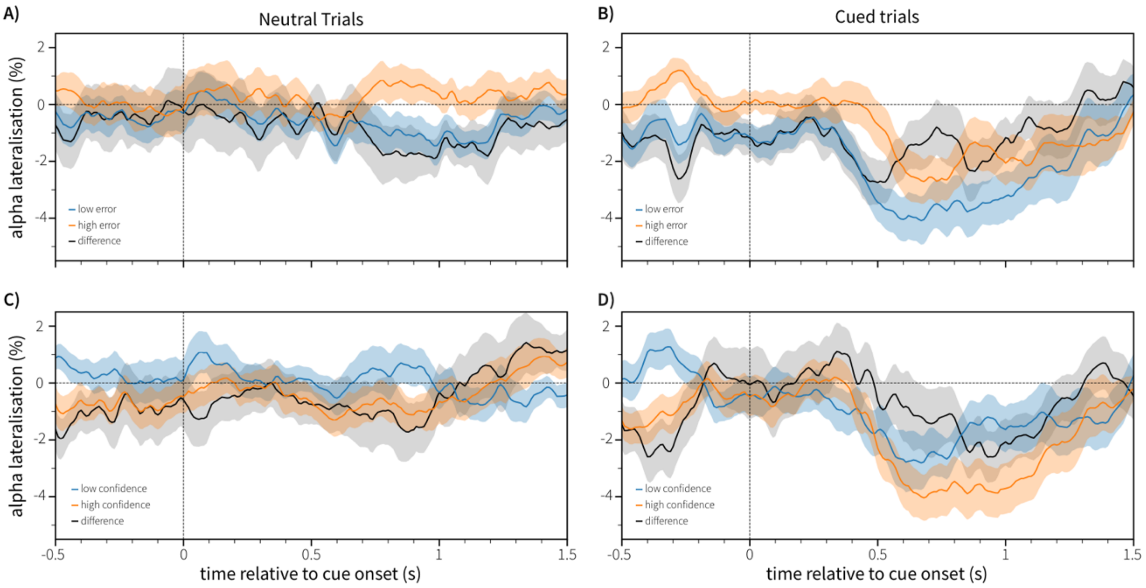

Appendix Figure 4 – Cue-induced alpha lateralisation relates to subsequent error and confidence in working-memory recall. Trials were binned based on a median split of within-participant participant performance across trials, separately for neutral (left column) and cued trials (right column). A) Median split over response error for neutral trials. B) Median split over response error for retrocued trials. C) Median split over confidence for Neutral trials. D) Median split over confidence for retrocued trials. Black lines represent the average within-participant difference between the blue and orange lines. Shaded regions represent the standard error of the mean.

Appendix 5 – Trial-to-trial changes in confidence are not driven by attentional changes at encoding

Modelling of behavioural responses highlighted that confidence changed systematically between trials depending on the accuracy of a confidence judgement the participant made – e.g. high degrees of overconfidence were associated with a subsequent lowering of confidence on the next trial. One candidate mechanism for these trial-wise effects is that attention is oriented differently depending on the feedback that participants receive. For example, when receiving feedback that participants performed much worse than expected (large error with high confidence), participants may subsequently refocus attention in order to improve performance on the next trial.

To test this, we tested whether posterior alpha power at encoding varied as a function of feedback on the previous trial. We fit a GLM to the data to model how alpha power at encoding on the current trial covaries with confidence error on the previous trial, while controlling for the effect of previous trial confidence (as the width of the reported confidence wedge also controls the maximum degree of confidence error). This uses the same regressor that behavioural analyses find relevant for

confidence updating between trials. Alpha power was baselined between 500 ms and 300 ms prior to array onset.

Here we see no significant effect of previous trial confidence error on posterior alpha power at encoding (Appendix figure 5, top panel). We also provide an alternative visualisation, where trials are instead split based on the categorical feedback presented on the previous trial (previously incorrect in red, and previously correct in purple). Here we again see no significant difference in posterior alpha power induced by the array based on previous trial feedback (the average within-participant difference is shown in the black line). This suggests that confidence error may not be used to re-orient attention on a trial based on recent history, but instead has some effect on confidence that is unlinked to attentional focus at encoding.

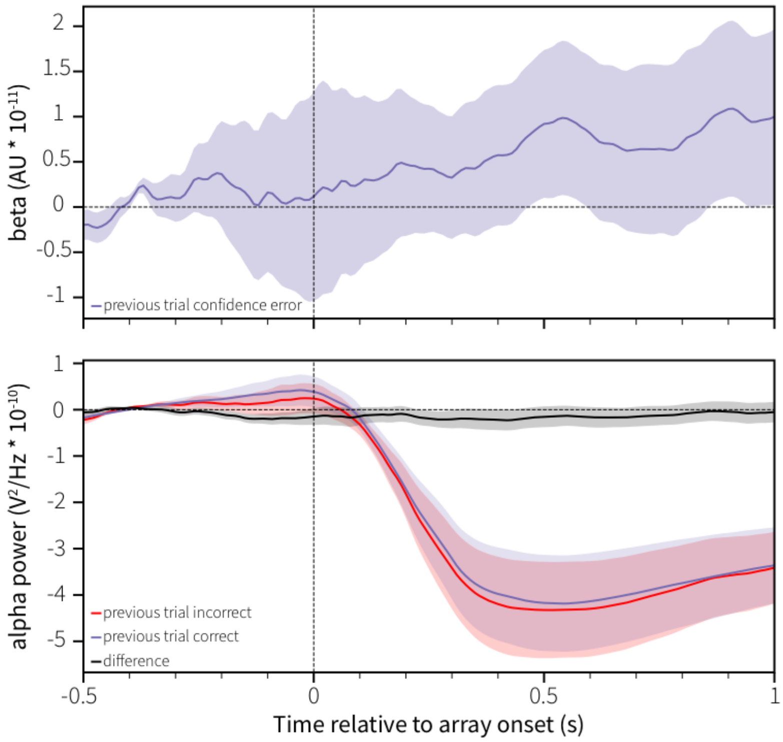

Appendix figure 5 – Confidence error is not systematically linked to induced alpha power on the proceeding trial. Top panel: output of a GLM analysis the association between array-induced alpha power and previous trial confidence error. No significant clusters emerge linking the degree of over- or under-confidence on a trial to attentional orienting at encoding on the subsequent trial, as indexed through posterior alpha power. Bottom panel: alternative visualisation where average alpha power at encoding is visualised separately for trials following negative feedback (red) or positive feedback (purple). The black line highlights the average within-participant difference between these trial types. No significant clusters emerge for the difference in alpha power between these trial splits. All shaded regions reflect the across-participant standard error of the mean, and solid lines reflect the participant average time course.
